# Induction of cuproptosis enhances sensitivity and overcomes resistance to osimertinib in lung cancer

**DOI:** 10.1038/s41392-025-02480-9

**Published:** 2025-11-26

**Authors:** Yi-Bo Gao, Jia-Ming Xie, Yan-Nan Yang, Xiao-Xiang Zhou, Jie He

**Affiliations:** 1 Central Laboratory & Shenzhen Key Laboratory of Epigenetics and Precision Medicine for Cancers, National Cancer Center/National Clinical Research Center for Cancer/Cancer Hospital & Shenzhen Hospital, Chinese Academy of Medical Sciences and Peking Union Medical College, Shenzhen, China; 2 Department of Thoracic Surgery, National Cancer Center/National Clinical Research Center for Cancer/Cancer Hospital, Chinese Academy of Medical Sciences and Peking Union Medical College, Beijing, China

**Keywords:** Drug development, Lung cancer, Drug screening

**Dear Editor**,

Drug resistance remains the most formidable challenge in lung cancer targeted therapy. Cuproptosis, a novel form of copper-dependent regulated cell death,^1,2^ has shown promise in overcoming radioresistance,^3^ but its potential in targeted therapy remains unexplored.

To clarify the adjunctive role of cuproptosis in targeted therapy, we designed an unbiased high-throughput screen of FDA-approved drugs (Fig. 1a). This approach led to the discovery that inducing cuproptosis through copper diethyldithiocarbamate (CuET) most strongly potentiated the efficacy of osimertinib among lung cancer drugs approved by the FDA. Further analysis of the synergy score between CuET and osimertinib was performed, and a highly synergistic effect was observed (Fig. 1a). Consistently, in the cuproptosis-induced group, the sensitivity of cells to osimertinib was increased, indicating that cuproptosis induction potentiates the anticancer efficacy of osimertinib (Fig. 1a). Further validation was performed via IncuCyte imaging, and CuET treatment strongly synergized with osimertinib in terms of growth inhibition (Fig. 1a). Taken together, these results highlight the potential of CuET in enhancing osimertinib efficacy.

**Fig. 1:**
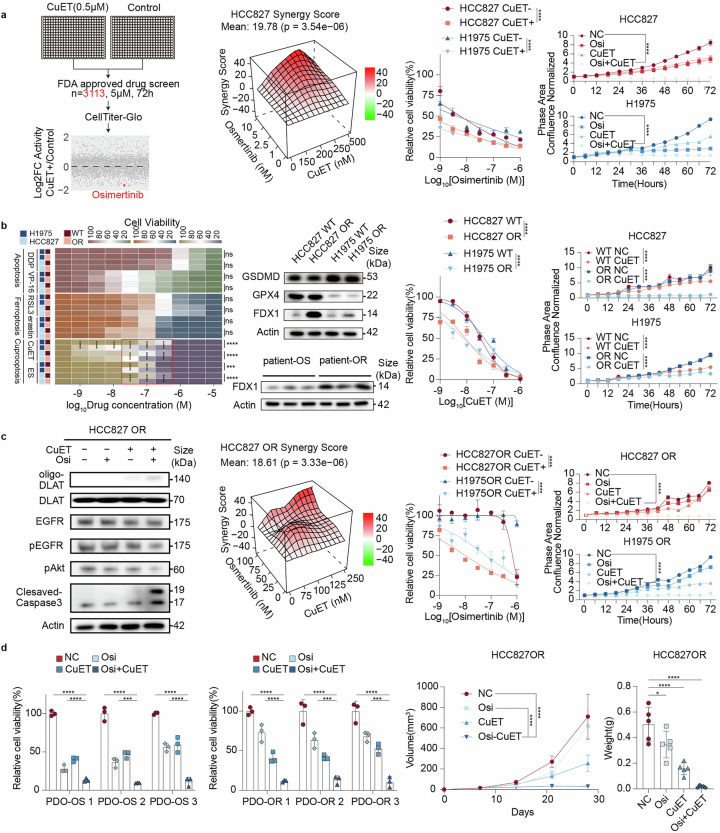
Induction of Cuproptosis Enhances Sensitivity and Overcomes Resistance to Osimertinib. **a** High-throughput screening revealed that cuproptosis synergizes with osimertinib. A total of 3113 FDA-approved compounds were screened at 5 μM for 72 h (far left). Synergy analysis of CuET (from 0.5 µM to 0.15 µM) and osimertinib (from 10 nM to 1 nM) was performed via the CellTiter-Glo assay (left). The cells were treated with seven concentrations of osimertinib (from 1 µM to 1 nM) for 72 h (n = 3 per dose) (right). H1975 and HCC827 cells were treated with CuET (0.25 µM), osimertinib (10 nM) or their combination and monitored with an IncuCyte live-cell analysis system (far right). **b** Osimertinib-resistant cells exhibit increased sensitivity to cuproptosis. Heatmap of cell viability upon treatment with various cell death inducers (far left). Protein levels in osimertinib-sensitive and osimertinib-resistant cells and patient samples (left). The cells were treated with seven concentrations of CuET (from 1 µM to 1 nM) for 72 h (n = 3 per dose) (right). H1975WT, H1975OR, HCC827WT and HCC827OR cells were treated with CuET (0.25 µM) and monitored with an IncuCyte live-cell analysis system (far right). **c** Protein levels in samples from HCC827OR cells treated with CuET (0.25 µM), osimertinib (10 nM) or their combination (far left). Synergy analysis of CuET (from 0.5 µM to 0.15 µM) and osimertinib (from 10 nM to 1 nM) was performed via the CellTiter-Glo assay (left). The cells were treated with CuET (0.25 µM) or 0.1% DMSO and seven concentrations of osimertinib (from 1 µM to 1 nM) for 72 h (n = 3 per dose) (right).H1975OR and HCC827OR cells were treated with CuET (0.25 µM) or 0.1% DMSO and osimertinib (0.1 µM) and monitored via an IncuCyte live-cell analysis system (far right). **d** Evaluation of the combination in physiologically relevant models. Relative viability of organoids derived from osimertinib-resistant patients treated with CuET (0.25 µM), osimertinib (10 nM, 0.1 µM) or their combination (left). Tumor volume and tumor weight in HCC827 OR xenografts treated with CuET (10 mg/kg), osimertinib (10 mg/kg), or their combination (right). (*p < 0.05, **p < 0.01, ***p < 0.001, ****p < 0.0001)

To further explore the role of cuproptosis in overcoming osimertinib resistance, we performed a second screen that evaluated which forms of regulated cell death, osimertinib-resistant cells, were selectively more sensitive than parental cells were (Fig. 1b). Osimertinib-resistant cell lines were generated via a standard prolonged-exposure method (Fig. 1b). In these induced resistant cells, the complementary screening identified cuproptosis as a top vulnerability. Moreover, among programmed cell death regulators, ferredoxin 1 (FDX1), the key driver of cuproptosis,^1^ was significantly upregulated in these osimertinib-resistant cells. In contrast, no significant differences in glutathione peroxidase 4 (GPX4, ferroptosis) or gasdermin D (GSDMD, pyroptosis) expression were detected (Fig. 1b). Notably, elevated FDX1 levels were also observed in samples from patients with osimertinib resistance (Fig. 1b), further supporting the role of cuproptosis in overcoming resistance. A cell viability assay confirmed enhanced cuproptosis-mediated cell death and increased CuET sensitivity in resistant lines, and IncuCyte assays corroborated these findings (Fig. 1b). Therefore, these findings collectively suggest that targeting cuproptosis may be a promising strategy to overcome osimertinib resistance.

Among the resistance mechanisms identified to date, bypass pathway activation is the most prevalent, accounting for approximately 46% of cases, and it often involves the phosphorylation of AKT.^4^ The combination treatment of cuproptosis induction and osimertinib markedly reduced p-AKT levels while increasing the levels of the cuproptosis marker oligomerized DLAT and the apoptosis marker cleaved caspase-3 (Fig. 1c). In osimertinib-resistant cell lines, the combination of CuET and osimertinib demonstrated high synergy scores (Fig. 1c). Moreover, the combination resulted in greater cell death induction and growth inhibition than did monotherapy in resistant cells, as shown by the results of cell viability and IncuCyte assays (Fig. 1c). Collectively, the above in vitro results suggest that cuproptosis could overcome osimertinib resistance possibly by reducing AKT phosphorylation, suggesting a promising strategy for osimertinib-resistant patients.

To further validate these findings, we tested the combination in physiologically relevant models. In patient-derived organoid models harboring EGFR mutations, including both osimertinib-sensitive and osimertinib-resistant samples, the combination outperformed either agent alone (Fig. 1d). In mouse xenograft models, the combination of CuET and osimertinib significantly reduced tumor volume and weight more effectively than monotherapy did (Fig. 1d).

In summary, both an unbiased drug screen and a complementary regulated cell death screen collectively identified cuproptosis as a candidate for overcoming osimertinib resistance. Notably, bypass pathway-mediated AKT activation represents one of the most prevalent yet insufficiently characterized mechanisms of resistance,^4^ and linking cuproptosis to this mechanism provides a rationale for combination therapy in this common yet underaddressed setting. With FDA-approved copper ionophore- and nanocarrier-based delivery systems under active development,^3,5^ these findings offer a feasible path toward clinical translation and warrant prospective trials to evaluate their safety and efficacy.

## Supplementary information

Supplementary materials

## Data Availability

All data underlying this study are provided in the article and its Supplementary Information. Additional data related to this study can be obtained from the corresponding author(s) upon reasonable request.

## References

[1] Tsvetkov, P. et al. Copper induces cell death by targeting lipoylated TCA cycle proteins. *Science* **375**, 1254–1261 (2022). 35298263 10.1126/science.abf0529 PMC9273333

[2] Yang, Z. et al. Hypoxia inducible factor-1α drives cancer resistance to cuproptosis. *Cancer Cell* **43**, 937–954.e9 (2025). 40054467 10.1016/j.ccell.2025.02.015

[3] Liao, Y. et al. A cuproptosis nanocapsule for cancer radiotherapy. *Nat. Nanotechnol.* **19**, 1892–1902 (2024). 39300223 10.1038/s41565-024-01784-1

[4] Cooper, A. J., Sequist, L. V. & Lin, J. J. Third-generation EGFR and ALK inhibitors: mechanisms of resistance and management. *Nat. Rev. Clin. Oncol.* **19**, 499–514 (2022). 35534623 10.1038/s41571-022-00639-9 PMC9621058

[5] Li, S. et al. Ultrasound-activated probiotics vesicles coating for titanium implant infections through bacterial cuproptosis-like death and immunoregulation. *Adv. Mater.* **36**, e2405953 (2024). 39101293 10.1002/adma.202405953

