# Supplementary material for: Induction of cuproptosis enhances sensitivity and overcomes resistance to osimertinib in lung cancer
Source: Signal Transduct Target Ther. 2025 Nov 26;10:390. doi: 10.1038/s41392-025-02480-9 (PMC12658086; doi:10.1038/s41392-025-02480-9)
Supplement: Supplementary file 1 — Supplementary materials [file 41392_2025_2480_MOESM1_ESM.docx]

Supplementary Materials for

Induction of Cuproptosis Enhances Sensitivity and Overcomes Resistance to Osimertinib in Lung Cancer

Yi-Bo Gao^1, 2#*^, Jia-Ming Xie^2#^, Yan-Nan Yang^1#^, Xiao-Xiang Zhou^2#^, Jie He^2*^

#These authors have contributed equally to this work

Correspondence to: [gaoyibo@cicams.ac.cn](mailto:gaoyibo@cicams.ac.cn), hejie@cicams.ac.cn

**This PDF file includes:**

Materials and Methods

Materials and Methods

Cell Lines and Culture Conditions

EGFR-mutant non-small cell lung cancer (NSCLC) cell lines NCI-H1975 and HCC827 were obtained from the American Type Culture Collection (ATCC). Cells were cultured in RPMI-1640 medium supplemented with 10% fetal bovine serum (Corning), 1% penicillin and streptomycin(Thermo) at 37°C with 5% CO2.

Establishment of Osimertinib-Resistant Cell Lines

Osimertinib-resistant cell lines (HCC827OR and H1975OR) were developed by gradually exposing wild-type cells to increasing concentrations of osimertinib over six months until stable resistance was achieved.

Compound Screening

A library of 3,113 FDA-approved compounds (5 μM, 72 hours) was screened to identify compounds that can be enhanced activity by cuproptosis. Compounds were tested in the presence or absence of copper diethyldithiocarbamate (CuET, TCI), cell viability was measured by CellTiter-Glo (Promega).

Synergistic Analysis

Cells were seeded in 96-well plates (3,000 cells per well) and treated after 24 hours with different concentrations of Osimertinib (MCE) and CuET, either alone or in combination, for 72 hours. Cell viability was assessed using CellTiter-Glo, and survival rates were calculated based on luminescence intensity.

Cell Viability Assay

Cells were seeded in 96-well plates (3,000 cells per well) and treated after 24 hours with drugs for 72 hours. After removing the medium with drugs, 10 μL of CCK-8 reagent(Dojindo) and 90 μL of fresh medium were added to each well. After 2 hours of incubation, absorbance was measured at 450 nm, and cell viability was calculated using GraphPad Prism software.

Cell Proliferation Incucyte live-cell analysis Assays

Real-time cell proliferation was monitored using the Incucyte live-cell analysis system (Essen BioScience). Cells were seeded at a density of 3,000 cells per well in 96-well plates and treated with 0.1% DMSO, CuET, Osimertinib, or their combination. Phase confluency was quantified over 72 hours.

Western Blot Analysis

Cells were lysed in RIPA buffer (Applygen) containing protease and phosphatase inhibitors (Thermo). Protein concentration was determined using the Pierce BCA Protein Assay Kit (Thermo). Equal amounts of protein were separated by SDS-PAGE and transferred to polyvinylidene difluoride (PVDF) membrane (Sigma-Aldrich). Membranes were incubated with indicated primary antibodies followed by HRP-conjugated secondary antibodies. Signals were visualized using an ECL detection system (Thermo).

Organoid Culture

Patient-derived organoids were established from samples of EGFR-mutant NSCLC patients. samples were dissociated using the Tissue Dissociation Kit (Miltenyi) and resuspended in Matrigel (Corning). After incubated at 37℃ for 15 min, organoids were cultured in advanced DMEM/F12 medium (Thermo) supplemented with B27, N2, GlutaMax , HEPES , Antibiotic–antimycotic, NAC, A83-01, Y-27632, SB202190, Nicotinamide and growth factors (R-spondin-1, Noggin, FGF10, FGF7 and EGF). The culture medium was replaced every 3 days, and organoids were passaged every 2 weeks.

Mouse Model

Female 6- to 8-week-old B6 mice were used for this study. Animal care and treatment followed institutional guidelines. A total of 5 × 10⁶ HCC827 OR cells in 100 µL PBS were subcutaneously injected into the right flank of six-week-old female BALB/c nude mice. Seven days post-implantation, mice were randomized into four groups (n = 5 per group) and treated with CuET (10 mg/kg), Osimertinib (10 mg/kg), or their combination via intraperitoneal injection. Tumor volume was measured weekly using calipers and calculated as: tumor volume = (length × width²)/2. After 28 days, mice were euthanized in accordance with institutional guidelines.
